# Meta-analysis of transcriptome reveals key genes relating to oil quality in olive

**DOI:** 10.1186/s12864-023-09673-y

**Published:** 2023-09-22

**Authors:** AliAkbar Asadi, Vahid Shariati, Soraya Mousavi, Roberto Mariotti, Mehdi Hosseini Mazinani

**Affiliations:** 1 National Institute of Genetic Engineering and Biotechnology (NIGEB), Shahrak-e Pajoohesh, Km 15, Tehran – Karaj Highway, PO Box 14965161, Tehran, Iran; 2 Institute of Biosciences and Bioresources, National Research Council, 06128 Perugia, Italy

**Keywords:** Olive, Meta-analysis, Olive oil, Oil quality, RNA-Seq, Oleic acid

## Abstract

**Background:**

Olive oil contains monounsaturated oleic acid up to 83% and phenolic compounds, making it an excellent source of fat. Due to its economic importance, the quantity and quality of olive oil should be improved in parallel with international standards. In this study, we analyzed the raw RNA-seq data with a meta-analysis approach to identify important genes and their metabolic pathways involved in olive oil quality.

**Results:**

A deep search of RNA-seq published data shed light on thirty-nine experiments associated with the olive transcriptome, four of these proved to be ideal for meta-analysis. Meta-analysis confirmed the genes identified in previous studies and released new genes, which were not identified before. According to the IDR index, the meta-analysis had good power to identify new differentially expressed genes. The key genes were investigated in the metabolic pathways and were grouped into four classes based on the biosynthetic cycle of fatty acids and factors that affect oil quality. Galactose metabolism, glycolysis pathway, pyruvate metabolism, fatty acid biosynthesis, glycerolipid metabolism, and terpenoid backbone biosynthesis were the main pathways in olive oil quality. In galactose metabolism, raffinose is a suitable source of carbon along with other available sources for carbon in fruit development. The results showed that the biosynthesis of acetyl-CoA in glycolysis and pyruvate metabolism is a stable pathway to begin the biosynthesis of fatty acids. Key genes in oleic acid production as an indicator of oil quality and critical genes that played an important role in production of triacylglycerols were identified in different developmental stages. In the minor compound, the terpenoid backbone biosynthesis was investigated and important enzymes were identified as an interconnected network that produces important precursors for the synthesis of a monoterpene, diterpene, triterpene, tetraterpene, and sesquiterpene biosynthesis.

**Conclusions:**

The results of the current investigation can produce functional data related to the quality of olive oil and would be a useful step in reducing the time of cultivar screening by developing gene specific markers in olive breeding programs, releasing also new genes that could be applied in the genome editing approach.

**Supplementary Information:**

The online version contains supplementary material available at 10.1186/s12864-023-09673-y.

## Background

Olive oil is a particularly important product because of its fatty acids and phenolic compounds, which are mainly responsible for the beneficial health aspects. Among these compounds, oleic acid and minor bioactive compounds have the highest impact on the quality and health effect of oil [1–3]. The International Olive Council (IOC) has also stated that oils with the highest levels of oleic acid are the most valuable nutritional products [4]. Currently, the cultivation areas and oil production increased in the world but only a few cultivars can yield consistently in the new environmental conditions and often changing negatively their quality profiles [5–7]. Therefore, according to the economic importance of olive oil, the quantity and quality of oil should be improved in parallel, based on international standards.

Several studies have noted that the main factors that influence olive oil quality are genotype, climatic and agronomic conditions, edaphic factors, and the technological method applied for oil extraction. Among these factors, genotype has a preponderant influence [8–16]. Besides studies show that 70% of the observed diversity in terms of fatty acid composition, phenolic compounds, bitterness or taste, and oil stability is genetically influenced [17–20].

One of the most important goals of RNA-seq experiments is to investigate changes in gene expression profiles under two or more different experimental conditions. The most of the RNA-seq studies performed in olives were related to the study of biotic and abiotic stresses [21, 22], micro-RNA identification [23], fruit developmental stages [24–27], and cold acclimation [28, 29]. Recently, some studies have focused on the role of environmental stresses such as high temperature and the altitude of cultivated areas on oil content and its quality using RNA-seq technique, but a limited number of these studies were directly related to the evaluation of oil quality [22, 30, 31]. In a study performed by Galla et al. [26], suppression subtractive hybridization (SSH) was used to isolate and identify a large set of genes that were differentially expressed at three different stages along olive fruit development. In another study conducted by Alagna et al. [24] differentially expressed genes involved in the metabolism of phenol and fatty acids at different stages of olive fruit development were identified. Moreover, in 2013, the olive cultivars' transcripts were used for de novo assembly and functional annotation [32]. In a study conducted by Parra et al. [33], the transcriptional regulation of the ripening process and activation of abscission zone were detected by RNA-seq. In 2016, the genome of Farga was sequenced and its annotation was identified by RNA-seq of leaf, root, and fruit samples [34]. Considering the importance of phenols in olive, in 2016, de novo transcriptome assembly was reported in olive fruit at different development stages and transcripts involved in flavonoid and anthocyanin pathways were identified [35]. In another study [36], the wild olive genome was sequenced and transcriptome analysis was performed to identify genes involved in oil biosynthesis. Recently, transcripts of all the enzymes in the biosynthetic pathway of tyrosol, hydroxytyrosol and secologanin, oleuropein’s precursor, were identified by the RNA-seq method in Koroneiki cultivar [37]. Furthermore, in 2019, targeted metabolome, Pacbio ISOseq transcriptome, and Illumina RNA-seq transcriptome were combined to investigate the relationship between phenols biosynthesis and differentially expressed genes during olive fruit development [27].

Technical variation in different experiments could affect the reproducibility of the research. Moreover, due to the cost of sequencing, RNA-seq experiments are mainly performed in a limited number of biological replicates, reducing the statistical power and the ability to detect and validate differences in gene expression. Accordingly, one of the most effective ways to improve reproducibility is to use multiple datasets through meta-analysis [38]. Therefore, re-analyzing existing data derived from several independent experiments can reveal new information and evaluate the most reliable key genes in a certain biosynthetic pathway. Meta-analysis of RNA-seq data can increase the speed of production of functional data related to the quality of olive oil and produce useful information. This study was conducted to analyze RNA-seq data obtained from multiple studies by meta-analysis approach to validate and identify key genes involved in the main metabolic pathways of oil quality.

## Results

### Meta-analysis results and comparison of different developmental stages

**Fig. 1:**
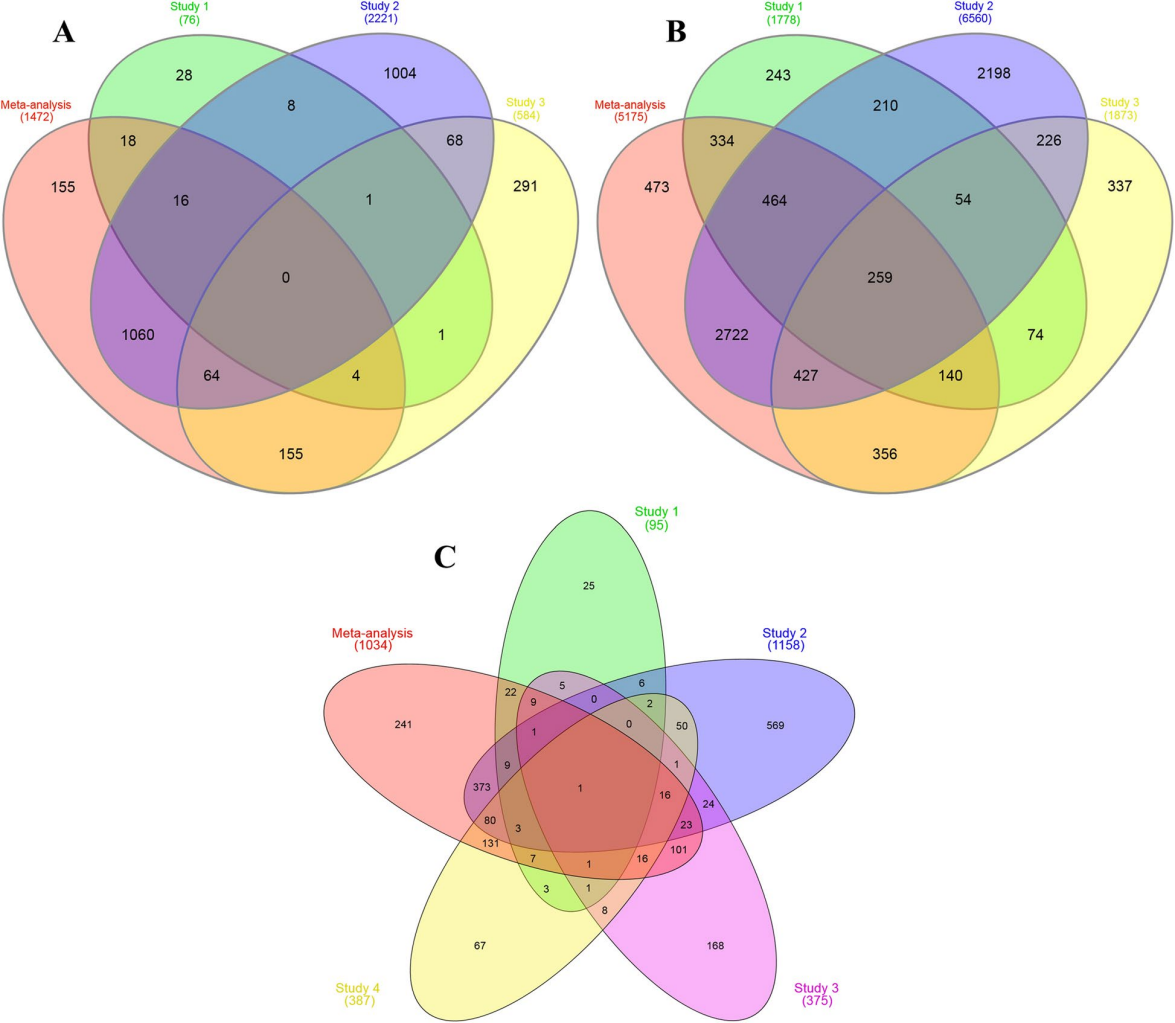
The Venn diagrams of overlapped and newly identified genes between different growth stages through meta-analysis. **A** Comparisons 1 (C1) *S1 vs. S2*; **B** C2: *S1 vs. S3*; **C** C3: *S2 vs. S3*. Study 1 referred to PRJNA514943; study 2: PRJNA524768; study 3: PRJNA638790; and study 4: PRJNA260808. The numbers in the Venn diagrams show the results of the meta-analysis, and the number of common and specific genes in the meta-analysis and other studies have been determined. The meta-analysis individuates 1472 differential expressed genes in S1 vs. S2 from which, 155 differential expressed genes were identified for the first time (**A**). The S1 vs. S3 has identified 5175 differential expressed genes, among them, 473 differential expressed genes have never been reported in the previous studies (**B**). The results of S2 vs. S3 identified 1034 differential expressed genes and in addition, 241 of them were identified for the first time (**C**)

The SRA and literature searches results showed that there were thirty nine experiments associated with the olive transcriptome from 2013 to 2022. By applying the filtration and quality control reported in the Methods section, four experiments had the ideal conditions to enter in our meta-analysis. The meta-analysis was performed to compare growth stages in pairs (comparison 1 (*C1): S1 vs S2*, comparison 2 (*C2): S1 vs S3*, and comparison 3 (*C3): S2 vs S3*) and the results of each comparison were shown as an independent Venn diagram (Fig. 1). The meta-analysis individuates 1472 differential expressed genes in *C1* comparison from which, 155 differential expressed genes were identified for the first time in the present study (Fig. 1A). The *C2* comparison has identified 5175 differential expressed genes (Fig. 1B), among them, 473 differential expressed genes have never been reported in the previous studies. The PRJNA260808 had only two developmental stages, S2 and S3, so only the C3 comparison was considered in this experiment. The results of *C3* comparison identified 1034 differential expressed genes (Fig. 1C), and in addition, 241 of them were identified for the first time.

The ratio of the identified differentially expressed genes in the meta-analysis to the total identified differential expressed genes were calculated as Integration-driven Discovery Rate (IDR) for each comparison. The IDR for *C1* was 10.53 and in *C2* and *C3* were 9.14 and 23.13, respectively.

### Identification of important metabolic pathways in oil quality

In order to identify essential and critical metabolic pathways in oil biosynthesis and quality compounds, two strategies were performed according to literature review (first step) and metabolic pathways (second step) described in Unver et al. [36]. In the first strategy, all the identified differential expressed genes in the meta-analysis were used for pathway enrichment and then important pathways selected. In the second strategy, to select critical metabolic pathways in oil quality, differential expression genes obtained from the meta-analysis were blasted against 2327 effective genes in oil biosynthesis [36]. The two steps led to enriching the pathways identified in each strategy. These steps were done separately for each comparison and selected common pathways between overall comparisons, pair comparisons, and specific to each comparison for the up and down-regulated genes (Tables 1 and 2). Sixteen metabolic pathways were common between all growth stages for the up-regulated genes (Table 1). The pathways of fatty acids biosynthesis and their elongation were in common between *C1* and *C2*. Furthermore, the most important individuated metabolic pathways were pentose phosphate, pyruvate metabolism, glycolysis/gluconeogenesis, fatty acid biosynthesis, fatty acid elongation, and biotin metabolism. The comparisons’ results also showed that the terpenoid compounds biosynthesis pathways were specific to *C2* including limonene and pinene degradation, diterpenoid biosynthesis, and monoterpenoid biosynthesis (Table 1).

**Table 1:** Identified metabolic pathways containing up-regulated differential expression genes. Each comparison (C) between two different growth stages (S) evaluated to identify the metabolic pathways. Growth stage less than 90 days after flowering (DAF) (X < 90; S1); S2 between 90 and 130 DAF (90 ≤ X < 130; S2); and finally, experiments with S3 of more than 130 DAF (X ≥ 130; S3)

Comparisons	Metabolic pathways
*C1: S1 vs S2* *C2: S1 vs S3* *C3: S2 vs S3*	1. Porphyrin and chlorophyll metabolism
	2. Glyoxylate and dicarboxylate metabolism
	3. Biosynthesis of unsaturated fatty acids
	4. Citrate cycle (TCA cycle)
	5. Fatty acid degradation
	6. Carotenoid biosynthesis
	7. Peroxisome
	8. Phenylpropanoid biosynthesis
	9. Glycerophospholipid metabolism
	10. Galactose metabolism
	11. alpha-Linolenic acid metabolism
	12. Glycerolipid metabolism
	13. Ubiquitin mediated proteolysis
	14. Sesquiterpenoid and triterpenoid biosynthesis
	15. Fatty acid metabolism
	16. Terpenoid backbone biosynthesis
*C1: S1 vs S2* *C2: S1 vs S3*	1. Cutin, suberine and wax biosynthesis
	2. Lipoic acid metabolism
	3. Biotin metabolism
	4. Pyruvate metabolism
	5. Proteasome
	6. Glycolysis / Gluconeogenesis
	7. Flavonoid biosynthesis
	8. Flavone and flavonol biosynthesis
	9. Fatty acid elongation
	10. Pentose phosphate pathway
	11. Fatty acid biosynthesis
*C2: S1 vs S3* *C3: S2 vs S3*	1. Brassinosteroid biosynthesis
	2. ABC transporters
*C2: S1 vs S3*	1. Limonene and pinene degradation
	2. Diterpenoid biosynthesis
	3. Monoterpenoid biosynthesis
*C3: S2 vs S3*	1. Linoleic acid metabolism

**Table 2:** Identified metabolic pathways containing down-regulated differential expression genes. Each comparison (C) between two different growth stages (S) evaluated to identify the metabolic pathways. Growth stage less than 90 days after flowering (DAF) (X < 90; S1); S2 between 90 and 130 DAF (90 ≤ X < 130; S2); and finally, experiments with S3 of more than 130 DAF (X ≥ 130; S3)

Comparison	Metabolic pathways
*C1: S1 vs S2* *C2: S1 vs S3* *C3: S2 vs S3*	1. ABC transporters
	2. alpha-Linolenic acid metabolism
	3. Glycerolipid metabolism
	4. Porphyrin and chlorophyll metabolism
	5. Glyoxylate and dicarboxylate metabolism
	6. Ubiquitin mediated proteolysis
	7. Biosynthesis of unsaturated fatty acids
	8. Pyruvate metabolism
	9. Citrate cycle (TCA cycle)
	10. Peroxisome
	11. Carotenoid biosynthesis
	12. Fatty acid degradation
	13. Glycolysis / Gluconeogenesis
	14. Pentose phosphate pathway
	15. Brassinosteroid biosynthesis
	16. Flavonoid biosynthesis
	17. Limonene and pinene degradation
	18. Phenylpropanoid biosynthesis
	19. Glycerophospholipid metabolism
	20. Monoterpenoid biosynthesis
	21. Fatty acid metabolism
	22. Galactose metabolism
	23. Terpenoid backbone biosynthesis
*C1: S1 vs S2* *C2: S1 vs S3*	1. Linoleic acid metabolism
	2. Flavone and flavonol biosynthesis
	3. Fatty acid elongation
*C2: S1 vs S3* *C3: S2 vs S3*	1. Biotin metabolism
	2. Fatty acid biosynthesis
*C2: S1 vs S3*	1. Sesquiterpenoid and triterpenoid biosynthesis

In the metabolic pathways containing down-regulated genes, 23 metabolic pathways were common between all developmental stages (Table 2).

### Grouping of identified metabolic pathways

The identified metabolic pathways were grouped according to the biosynthetic cycle of fatty acids and factors affecting oil quality, to provide more details about the most important identified pathways. In the study of oil biosynthesis, attention should be paid to the three key stages of carbon supply in the skeleton of triacylglycerols including the production of fatty acids, the assembly of fatty acids, and finally the formation of complex lipids [39].

I. Pathways related to carbon source: In the first group, there are metabolic pathways that supply carbon for production of fatty acids, and the pathway of galactose metabolism has been identified in this group (Fig. S1A and B - Additional file 1). There are several sources of carbon in olive tree [39], which are A) Leaves photosynthesis (transfer of photosynthetic products from leaves to other organs) B) Raffinose family oligosaccharides (making of sugar alcohols and oligosaccharides such as mannitol, raffinose, and stachyose along with other photosynthetic materials in separate reactions), and C) Fruit photosynthesis. In olive, biosynthesis of mannitol and sorbitol, which are sugar alcohol, is present, and this pathway was also observed in the *Apiaceae* family [40]. In addition, the biosynthesis of oligosaccharides such as stachyose and raffinose, which are found in the legumes family, was also observed in olives [39]. Therefore, it is possible to confirm that making sugar alcohols and oligosaccharides, as observed in other plant families, can be a reliable source of carbon also in olives.
II. Pathways related to Acetyl-CoA: In the second group, the presence of acetyl-CoA along with two enzymes acetyl-CoA carboxylase (ACCase) and fatty acid synthase (FAS) is necessary to start the biosynthesis of fatty acids [39]. It was stated that a stable and rapid pathway for the supply of acetyl-CoA is glycolysis and pentose phosphate pathways using pyruvate by the activity of the enzyme pyruvate dehydrogenase (AID in 2012). In the present study, the key metabolic pathways identified for acetyl-CoA biosynthesis were glycolysis (Fig. S2A and B - Additional file 2) and pyruvate metabolism (Fig. S3A and B - Additional file 3).
III. Pathways related to fatty acids: In the third group, biosynthesis of the carbon chain of fatty acids, the key metabolic pathways of fatty acid biosynthesis, fatty acid elongation, and biotin metabolism were individuated in the present study. Metabolic pathway glycerolipid metabolism was identified as a key metabolic pathway in the assembling of fatty acids and the formation of complex lipids. In addition, two metabolic pathways alpha-linolenic acid metabolism and linoleic acid metabolism were identified as important pathways in the production of by-products of fatty acid biosynthesis.
IV. Pathways related to non-fatty acids components (minor compound): The fourth group was related to metabolic pathways that have a direct impact on the quality of the oil and its taste. In this group, six key metabolic pathways were observed: terpenoid backbone biosynthesis, phenylpropanoid biosynthesis, flavonoid biosynthesis, limonene and pinene degradation, monoterpenoid biosynthesis, and carotenoid biosynthesis.

In order to more closely examine the identified enriched metabolic pathways, the most important enriched metabolic pathways affecting oil quality were identified in each group and have been investigated independently. Finally, the key genes affecting each metabolic pathway have been identified and introduced (Table 3). Also, the accession numbers of the identified up- and down-regulated genes in each of the investigated comparisons are provided in Additional file 4, Table S2.

**Table 3:** Summary of identified key genes in the main pathways for olive oil quality

**Group**	**Pathway**	**Important genes in the meta-analysis**
Carbon source	Galactose metabolism (oeu00052)	Raffinose synthase
		Galactinol synthase
		UDP-glucose epimerase
		Alpha-galactosidase
Acetyl-CoA biosynthesis	Glycolysis pathway (oeu00010)	Phosphoglucomutase
		6-phosphofructokinase
		Fructose-bisphosphate aldolase
		Triose-phosphate isomerase
		Glyceraldehyde-3-phosphate dehydrogenase
		Phosphoglycerate mutase
		Pyruvate kinase
		Pyruvate dehydrogenase beta subunit
		Pyruvate dehydrogenase (pdhC)
		Pyruvate decarboxylase
		Alcohol dehydrogenase
		Aldehyde dehydrogenase
		Acetyl-CoA synthetase
	Pyruvate metabolism (oeu00620)	Phosphoenol pyruvate carboxylase
Fatty acid biosynthesis and Glycerolipid production	Fatty acid biosynthesis (oeu00061)	Acetyl CoA carboxylase
		FabH (beta-ketoacyl-[acp] synthase III)
		FabF (beta-ketoacyl-[acp] synthase II)
		FabG (beta-ketoacyl-[acp] (ACP) reductase)
		FabI (enoyl-[acp] reductase)
		Stearoyl-[acp] 9-desaturase (SAD)
		Fatty acyl-ACP thioesterase A (FATA)
	Biosynthesis of unsaturated fatty acid (oeu01040)	Fatty acid desaturase 2 (FAD2)
	Fatty acids degradation (oeu00071)	Long-chain acyl-CoA synthetase (LACS)
		Acyl-CoA oxidase
		Enoyl-CoA hydratase (ECHS)
		Beta-hydroxyacyl dehydrogenase
		Long-chain-3-hydroxyacyl-CoA dehydrogenase
		Acetyl-CoA acyltransferase
	Glycerolipid metabolism (oeu00561)	Glycerol kinase
		Aldehyde dehydrogenase
		Alcohol dehydrogenase
		Lysophosphatidic acid acyltransferase
		Phosphatidate phosphatase
		Diacylglycerol kinase
		Phospholipid:diacylglycerol acyltransferase (PDAT)
		Diglyceride acyltransferase (DGAT)
		Acylglycerol lipase
Secondary metabolites	Terpenoid backbone biosynthesis (oeu00900)	1-deoxy-D-xylulose-5-phosphate synthase (DXS)
		2-C-methyl-D-erythritol 4-phosphate Cytidylyltransferase (CDPMES)
		4-hydroxy-3-methylbut-2-en-1-yl diphosphate reductase (HMBPPR)
		4-diphosphocytidyl-2-C-methyl-D-erythritol kinase (CDPMEK)
		Hydroxymethylglutaryl-CoA synthase (HMGCS)
		Mevalonate kinase (MVAK)
		Hydroxymethylglutaryl-CoA reductase (HMGCR)
		Phosphomevalonate kinase (MVAPK)
		Diphosphomevalonate decarboxylase (MVAPPD)
		Isopentenyl-diphosphate Delta-isomerase
		Dimethylallyl transtransferase
		Farnesyl-diphosphate synthase
		Geranylgeranyl diphosphate synthase

## Discussion

In olive, breeding programs last about 30 years on average [41, 42], while the timing for the selection of new cultivars in other fruit crops has been greatly reduced, also by the application of new efficient genomic tools [43–45]. In cultivated olives (*Olea europaea* subsp. *europaea* var. *europaea*), the crossbreeding activities have been delayed by the particularly long generation time [46], the extended juvenile phase and the high demanding nursery practices. This work provides the best candidate genes to construct markers for a fast and reliable genotyping of olive cultivars for their oil quality, offering great opportunities to rapidly screen and planning inter-varietal crosses, and reducing the time for seedling selection. Furthermore, the newly detected candidate genes could be applied as a source for interested traits in genome editing approaches.

The IDR index demonstrated that meta-analysis has good power to identify new differentially expressed genes. Therefore, the low value of IDR indicates a subtly increase in the power of meta-analysis compared to the results obtained in the previously published independent studies [47]. In addition, the decrease in the number of new differentially expressed genes in the meta-analysis compared to the total genes that were significantly different in comparison between the two developmental stages indicated that the meta-analysis was more efficient than independent studies and provided solid results.

### Galactose metabolism pathway (oeu00052)

**Fig. 2:**
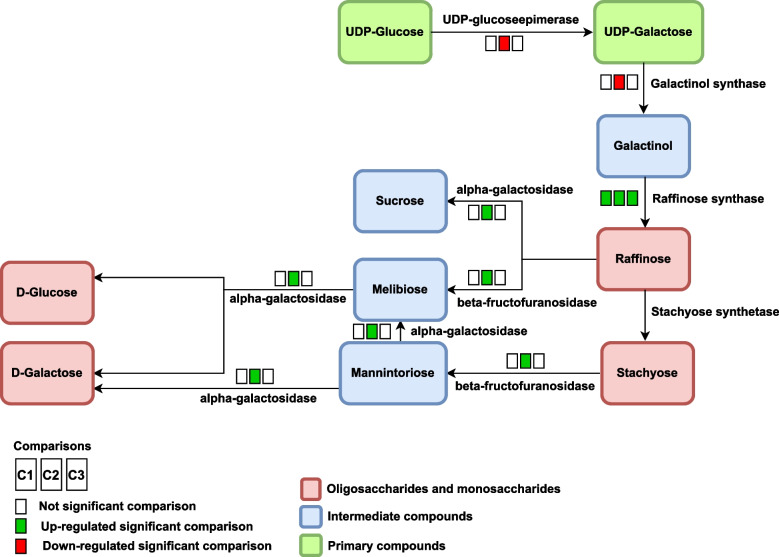
The biosynthesis of raffinose family oligosaccharides in the galactose metabolism pathway. The first square show the results of comparison 1 (*C1*: S1 *vs*. S2) and second and third squares show comparison 2 (*C2*: S1 *vs*. S3) and comparison 3 (*C3*: S2 *vs*. S3) respectively. For differential compounds, boxes in red represent down-regulated genes in each comparison, boxes in green represent the up-regulated genes, and boxes in white represent no significant change

In olives, sugars are one of the main components that play a role in providing energy and function as precursors to oil biosynthesis. In the *Oleaceae*, the olive tree is a unique species among others in terms of the synthesis of the raffinose family of oligosaccharides [39, 40]. Galactinol biosynthesis is one of the most essential step in the initiation of raffinose biosynthesis (Fig. 2). Regarding metabolic pathway of galactose in the present study showed that an increase in expression of raffinose synthase (EC: 2.4.1.82), in all growth stages, can increase the biosynthesis of raffinose. This enzyme produces raffinose by transferring the galactosyl moiety from galactinol to sucrose, allowing raffinose to be converted to stachyose [48]. The activity of the raffinose synthase enzyme showed that the production of this enzyme increases 5 days after flowering, reaches its maximum level after 15 days, and has a constant trend until the end of the growth stages, in soybean [49]. Increased expression of this enzyme at all stages of development can be due to the high demand of the fruit for energy and carbon source. Furthermore, the conversion of UDP-Galactose to galactinol is performed by the enzyme galactinol synthase (EC: 2.4.1.123), which is known as the starting point for raffinose biosynthesis in the galactose metabolic pathway [48]. The results of the present study showed that the expression of the galactinol synthase reduced in the growth stage of S3 as well as the decrease in raffinose production. In Le et al. [50] study, which was conducted in soybeans, it is shown that knockout of galactinol synthase encoding genes leads to a decrease in the production of raffinose, oligosaccharides family, and in addition to changes in the amount of carbohydrates, it also caused the changes in the amount of protein and fat in soybeans. Our results also showed that the expression of UDP-glucose epimerase (EC: 5.1.3.2) decreased in the growth stage of S3. The enzyme UDP-glucose epimerase converts UDP-glucose to UDP-galactose to be used for galactinol production. Probably reducing the expression of these two enzymes in the developmental stage S3 reduced the production of galactinol, followed by raffinose.

Galactinol synthase, in the metabolic pathway of galactose, is a stress-related gene. Olive tree under abiotic stress produce antioxidants and osmolytes such as sugar alcohols, raffinose oligosaccharides. Therefore, raffinose should not have a direct effect on the initial tolerance of abiotic stresses, but it can help protect the plant against oxidative damage [28, 49]. Another important gene is the enzyme alpha-galactosidase (EC: 3.2.1.22). Our results showed that the expression of this enzyme increases in the growth stage of S3 and indicates an increase in the cell's demand for simple carbohydrates such as glucose and galactose [51]. This enzyme can also convert melibiose to galactose and glucose so that they can be used in oil biosynthetic pathways. Finally, in accordance with Conde et al. [51], this enzyme, with its hydrolyzing role, could convert complex oligosaccharides and carbohydrates into simple forms so that they can be easily delivered to the cell.

### Glycolysis pathway (oeu00010) and pyruvate metabolism pathway (oeu00620)

The metabolic pathway of glycolysis and pentose phosphate are the main sources for the production of acetyl-CoA from pyruvate [52]. In addition, in olives, the breakdown of glucose through the metabolic pathway of glycolysis and the production of acetyl-CoA from pyruvate in plastid is common [53]. Increased expression of glycolysis metabolic pathway genes indicated that this pathway is the most active in growth stages S2 and S3. According to the results of the current meta-analysis, the glycolysis pathway has more enriched genes (21 enriched genes) than the pentose phosphate pathway (11 enriched genes) that are effective in pyruvate production (Table S1 - Additional file 5).

**Fig. 3:**
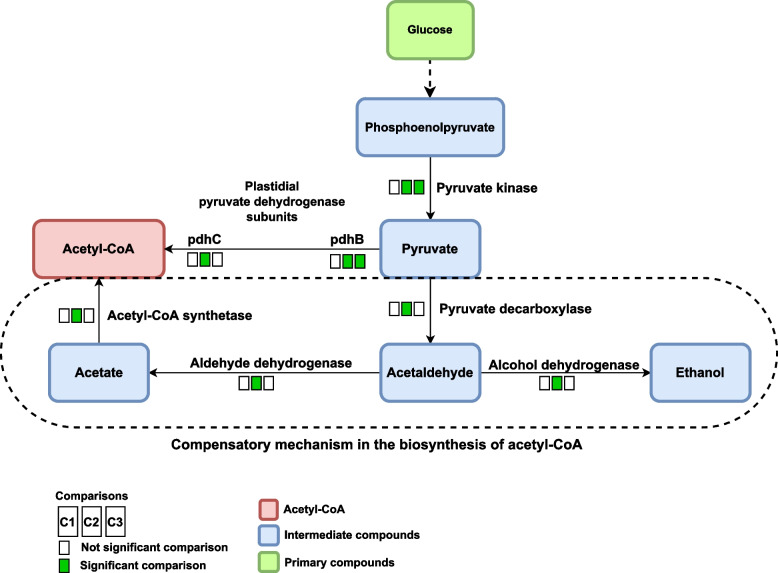
Acetyl-CoA production from pyruvate through different precursors. The first square show the results of comparison 1 (*C1*: S1 *vs*. S2) and second and third squares show comparison 2 (*C2*: S1 *vs*. S3) and comparison 3 (*C3*: S2 *vs*. S3) respectively. For differential compounds, boxes in green represent up-regulated genes in each comparison and boxes in white represent no significant change

In the metabolic pathway of glycolysis for pyruvate biosynthesis from alpha-D-Glucose1-phosphate enzymes are involved the phosphoglucomutase (EC: 5.4.2.2), 6-phosphofructokinase (EC: 2.7.1.90 and EC: 2.7.1.11), fructose-bisphosphate aldolase (EC: 4.1.2.13), triose-phosphate isomerase (EC: 5.3.1.1), glyceraldehyde-3-phosphate dehydrogenase (EC 1.2.1.12), phosphoglycerate mutase (EC: 5.4.2.11 and EC: 5.4.2.12), and pyruvate kinase (EC: 2.7.1.40) and the expression of them increased in developmental stages S2 and S3. Galla et al. [26] found that the expression of 6-phosphofructokinase, glyceral-dehyde-3-phosphate dehydrogenase, phosphoglycerate kinase, phosphopyruvate hydratase, and pyruvate kinase increased from 30 to 90 days after flowering and their expression decreases from 90 to 130 days after flowering. Since the metabolic pathway of glycolysis to produce pyruvate has ten steps, the enzymes 6-phosphofructokinase, glyceraldehyde-3-phosphate dehydrogenase, and pyruvate kinase are the key enzymes of this pathway that play an important role in the biosynthesis of acetyl-CoA and followed by fatty acids. The study by Guerin et al. [54] on the gene co-expression network of the oil biosynthesis pathway in the palm indicated that the enzymes hexokinase, fructokinase, 6-phosphofructokinase, glyceraldehyde-3-phosphate dehydrogenase, and pyruvate kinase work directly with genes involved in fatty acids biosynthesis. The phosphoglucomutase enzyme, which was identified in our meta-analysis and was not discussed in previous studies, can play a key role in providing carbon sources to produce fatty acids. By converting Glucose 1-phosphate to Glucose 6-phosphate, this enzyme produces Glucose 6-phosphate, which is an effective and important precursor to produce starch and fatty acids. In the Arabidopsis, phosphoglucomutase mutants showed that the percentage of oil in the mutants of this gene decreases by about 40% compared to the wild genotype [55]. Therefore, one of the consequences of blocking the conversion of Glucose 1-phosphate to Glucose 6-phosphate in the phosphoglucomutase mutant was the restriction of carbon flow towards the synthesis of fatty acids, which at least partially led to a decrease in oil content. Another important enzyme in the glycolysis pathway, identified in the current meta-analysis and has never been reported in olive, is fructose-bisphosphate aldolase enzyme. This enzyme is located on upstream of fatty acid biosynthesis and is involved in the production of two important metabolites, dihydroxyacetone phosphate and glyceraldehyde 3-phosphate, which are also important in oil biosynthesis [56]. The study of the relationship between this enzyme and oil biosynthesis in *Camellia oleifera* showed that the mRNA level of this enzyme had a positively correlation with the mRNA level of the stearoyl-[acp] 9-desaturase (SAD) enzyme and it can affect the amount and quality of the oil. In the research of Zeng et al. [57], it has been suggested that fructose-bisphosphate aldolase and SAD enzymes are two important factors for determining the amount of oil in *Camellia oleifera* because fructose-bisphosphate aldolase enzyme controlled the production of important intermediate compounds in oil biosynthesis and SAD enzymes regulated oleic acid production. The results of our meta-analysis showed that the biosynthesis pathway of acetyl-CoA from pyruvate is a fast and stable pathway. Furthermore, the enzymes effective in the production of pyruvate start their activity before the accumulation of oil, from the S2 stage, and continue the biosynthesis of pyruvate to the S3 development stage. After the production of pyruvate, it is converted to acetyl-CoA by the activity of plastid pyruvate dehydrogenase (EC: 1.2.4.1 and EC: 2.3.1.12). The results of the present study showed that the pyruvate dehydrogenase beta subunit (pdhB) (EC: 1.2.4.1) started its activity from the growth stage of the S2 and continued in the S3, while the other subunit of pyruvate dehydrogenase (pdhC) (EC: 2.3.1.12) was active only in the developmental stage of S3. On the other hand, the results showed that the expression of pyruvate kinase (EC: 2.7.1.40) and the pdhB decreased in the growth stage of the S3. Therefore, according to the results, the highest rate of pyruvate accumulation is observed in the S2 and only the pdhC is active in the S3 stage. In addition, to decrease the pdhB expression in the S3, the expression of pyruvate decarboxylase (EC: 4.1.1.1) and alcohol dehydrogenase (EC: 1.1.1.2) increased and pyruvate is converted to acetaldehyde and ethanol. Our results also showed that in the S3, olive used other paths to produce acetyl-CoA and convert acetaldehyde to acetate by the enzyme aldehyde dehydrogenase (EC: 1.2.1.3) and acetate to acetyl-CoA by the enzyme acetyl-CoA synthetase (EC: 6.2.1.1) (Fig. 3). According to the obtained results, the expression of two enzymes, aldehyde dehydrogenase and acetyl-CoA synthetase, increased only in the S3 growing stage. Research by Guerin et al. [54] on the gene co-expression network of the palm oil biosynthesis showed that the two enzymes aldehyde dehydrogenase and acetyl-CoA synthetase were present in the mesocarp expression network and indirectly affect the biosynthesis of fatty acids. The results of the present study also showed that the expression of acetyl-CoA synthetase decreased in the growth stage S2 and only in the S3 its expression increased, which could play a compensatory mechanism in the biosynthesis of acetyl-CoA, therefore, the high demand of energy of olive fruit during these stages [26].

The results also show that increasing the expression of phosphoenol pyruvate carboxylase (EC: 4.1.1.31) enzyme in the growth stages S2 and S3 in the pyruvate metabolism pathway (oeu00620), causes the conversion of oxaloacetate to phosphoenol pyruvate and finally to pyruvate and acetyl-CoA. The presence of the phosphoenol pyruvate carboxylase in olive mesocarp, which is present in CAM and C4 plants, helps as an additional pathway along with photosynthesis in carbon supply and pyruvate production [24, 26]. Therefore, the plastidial production of acetyl-CoA through the metabolic pathways of glycolysis and pyruvate metabolism is a rapid and stable pathway to begin the biosynthesis of fatty acids and other pathways of acetyl-CoA supply outside the plastids (production of acetyl-CoA from acetate) could contribute to the supply of acetyl-CoA during the fruit development stage.

### Fatty acid biosynthesis pathway (oeu00061)

The initiation step in the fatty acid biosynthesis involves irreversible conversion of acetyl-CoA to malonyl-CoA, which is done by the enzyme acetyl-CoA carboxylase (acetyl-CoA carboxylase biotin carboxyl carrier protein; ACCase) and is regulatory enzyme in the fatty acid biosynthesis [39, 51]. According to the meta-analysis results, the ACCase (EC: 6.4.1.2) enzyme is synthesized via the biotin metabolic pathway (oeu00780) (Fig. S4A and B - Additional file 6). The expression of genes responsible for producing this enzyme increases in the S2 developmental stage, followed by decreases in the S3 stage. Additionally, the findings suggest that the ACCase enzyme expression increases during the S3 growth stage, as observed in the fatty acid biosynthesis pathway (Fig. S5A and B - Additional file 7) and the pyruvate metabolism pathway.

The production of malonyl-CoA is known to be an essential step in the biosynthesis of fatty acids because malonyl-CoA is a precursor to the enzyme fatty acid synthase (FAS) [51]. The results showed that FabH (beta-ketoacyl-[acp] synthase III; KAS III) (EC: 2.3.1.180) is also important in the early stage of biosynthesis of fatty acids and its expression increased in growth stages S2 and S3. Increasing the activity of FabH enzyme at the beginning of the biosynthetic pathway of fatty acids helps the accumulation of acetoacetyl-[acp] to be used in the elongation phase. The ACCase enzyme and FabH are necessary genes for the de novo formation of acyl chains in the palm plastid [54].

**Fig. 4:**
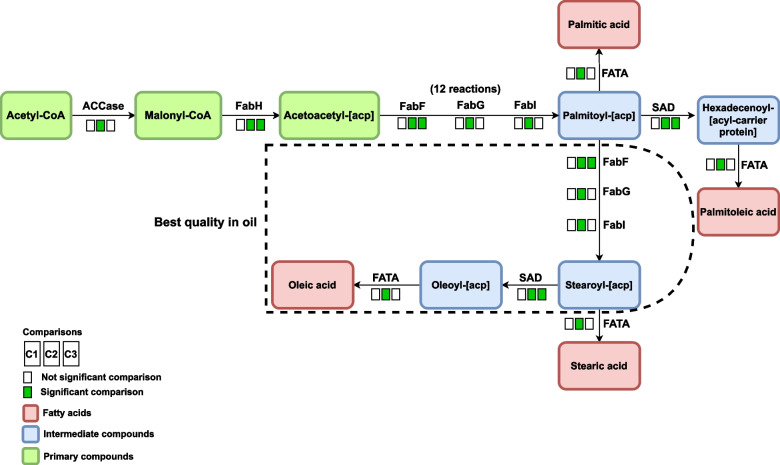
Fatty acid biosynthesis highlighting the main compounds involved in olive oil quality. The first square show the results of comparison 1 (*C1*: S1 *vs*. S2) and second and third squares show comparison 2 (*C2*: S1 *vs*. S3) and comparison 3 (*C3*: S2 *vs*. S3) respectively. For differential compounds, boxes in green represent up-regulated genes in each comparison and boxes in white represent no significant change

According to the obtained results, the elongation stage of fatty acids consists of 12 independent reactions and the present meta-analysis detected that three enzymes FabF (beta-ketoacyl-[acp] synthase II, KAS II) (EC: 2.3.1.179), FabG (beta-ketoacyl-[acp] (ACP) reductase, KAR) (EC: 1.1.1.100), and FabI (enoyl-[acp] reductase; ENR) (EC: 1.3.1.9 and EC: 1.3.1.10) participate in the production of palmitoyl-[acp] which are important enzymes in FAS complex enzyme (Fig. 4). Furthermore, the results show that the expression of FabF enzyme increases in growth stages S2 and S3 while the expression of FbaG and FabI enzymes increased only in growth stage S3. According to the obtained results, the activity of FabF enzyme increased from the growth stage of S2, which showed that the continuous production of primary compounds and precursors for initiating reactions is done with the activity of this enzyme. The elongation phase of fatty acids ends with the production of the palmitoyl-[acp] and entry of palmitoyl-[acp] into subsequent pathways is known as the termination step in fatty acid biosynthesis. The palmitoyl-[acp] can enter in two different ways and produce the next fatty acids:

I. In the first pathway, palmitoyl-[acp] is converted to palmitic acid by the enzyme fatty acyl-ACP thioesterase A (FATA) (EC: 3.1.2.14) or it can convert to its unsaturated form by the activity of the SAD enzyme and then is converted to palmitoleic acid by FATA enzyme (Fig. 4). The results of the present study showed that the activity of SAD enzyme increases in growth stages S2 and S3, probably resulting palmitoleic acid production rather than palmitic acid.
II. In the second pathway, FabF is a key enzyme that has a critical role in oil quality and also C16/C18 fatty acid ratio is determined [39]. Studies in oil palm show that enzyme FabF has a negative correlation with C16:0 fatty acids, and it plays an important role in determining the percentage of C16:0 fatty acids [54]. Together with FabF, an increase of SAD enzyme expression in stages S2 and S3 could convert stearoyl-[acp] to oleoyl-[acp] with an unsaturated bond. The result of the meta-analysis demonstrated high expression of SAD (EC: 1.14.19.2) observed in S2 and S3 and the activity of SAD enzyme started from the growth stage S2 and continues in the S3 stage, which can increase the production of oleoyl-[acp] and improve the quality of olive oil. Our results showed that the expression of SAD enzyme decreased only in stage S3 and, accordingly, the conversion of saturated to unsaturated fatty acids is done in S2. Therefore, if the activity of SAD decreases, the conversion of stearoyl-[acp] to oleic acid will also decrease and stearoyl-[acp] will be transformed to stearic acid. Gene silencing for SAD in *Chlamydomonas reinhardtii* showed the mRNA levels of the enzyme were decreased after the silencing construct was induced and the reduction in SAD mRNA caused in a doubling of the stearic acid content in triacylglycerol molecules [58]. Increasing the expression of FATA in S3 causes the ACP agent to be isolated from oleoyl-[acp] and oleic acid is produced and could convert stearoyl-[acp] to stearic acid (Fig. 4). The thioesterase enzyme has two forms that FATA preferentially isolated ACP moiety from unsaturated fatty acid [39]. The RNAi technique in the soybean showed down-regulation of FATB, other form of the thioesterase, which cause the synthesis of oleic acid in high levels and the reduction of palmitic acid content [56]. Also, the results of studies in palm showed SAD and FATA enzymes have a positive correlation and a negative correlation with saturated C18 fatty acids [54]. Therefore, the second pathway is more suitable for producing of high oleic acid and, therefore, improving oil quality.

Oleic acid produced at the end of fatty acid biosynthesis steps can be converted into linoleic acid by fatty acid desaturases (FAD) enzymes [56]. For this reason, the ratio of oleic acid to linoleic acid is considered one of the indicators related to olive oil quality. The results of the meta-analysis show that the expression of the fatty acid desaturase 2 (EC: 1.14.19.6 and EC: 1.14.19.22) (FAD2) enzyme decreases in S2 and S3 growth stages, and then the conversion of oleic acid to linoleic acid will also decrease (Fig. S6A and B - Additional file 8). Hernández et al. [59] identified two genes (OeFAD2-1 and OeFAD2-2) responsible for microsomal oleate desaturases in olives. OeFAD2-2 was found to be the primary gene determining linoleic acid content in the olive mesocarp and virgin olive oil [60, 61]. On the other hand, due to the increase in the expression of the SAD enzyme in the growth stages S2 and S3, and the decrease in the expression of the FAD2 enzyme in these growth stages, it helps to increase and maintain the level of oleic acid production. A study conducted by Contreras et al. [62] on the Leccino and Coratina cultivars, investigating the expression of SAD and FAD genes in various olive tissues. The research found that the OeSAD1 and OeSAD2 genes had the highest expression in the fruit, while the OeFAD expression was the lowest in this tissue [62]. It was also discovered that in both genotypes, the expression of OeSAD1 and OeSAD2 was at its highest during and after the pit-hardening period when oil accumulation in the fruit mesocarp was rapidly increasing. Conversely, the expression level of both OeFAD2 genes was almost negligible during fruit ripening [62]. In order to improve the nutritional properties and oxidative stability of plant oils, efforts have been made in oilseeds to obtain high oleic lines by reducing FAD2 expression and/or activity. This is because plant oils with a higher oleic/linoleic ratio are considered to have superior nutritional properties [63].

The results showed that the expression of long-chain acyl-CoA synthetase (LACS) (EC: 6.2.1.3) enzyme increases in S3 stage and a CoA group is added to the palmitic acid and finally forming long-chain acyl-CoA. In other words, the production of long-chain acyl-CoA can also be the beginning of the fatty acids degradation via beta-oxidation with LACS (oeu00071) (Fig. S7A and B - Additional file 9). This stage is the beginning of the path in the activation of long-chain fatty acids for synthesis of cellular lipids, fatty acid degradation via beta-oxidation, and cuticle development [64, 65]. Using palmitic acid in the production of long-chain fatty acids can be effective in improving oil quality. In a study conducted by Ramírez-Tejero et al. [66], the LACS2 gene was identified as one of the essential fruit-specific genes that encode long-chain acyl-CoA synthetase 2 enzyme which activated long-chain fatty acids for degradation via beta-oxidation. The meta-analysis indicated that five main enzymes involved in fatty acid peroxisomal beta-oxidation being, acyl-CoA oxidase (EC: 1.3.3.6), Enoyl-CoA hydratase (ECH) (EC: 4.2.1.17), beta-hydroxyacyl dehydrogenase (HADH) (EC: 1.1.1.35), long-chain-3-hydroxyacyl-CoA dehydrogenase (LCHAD) (EC: 1.1.1.211), and acetyl-CoA acyltransferase (fadA) (EC: 2.3.1.16). The results of the present study showed an up-regulation of ECH, LCHAD, and fadA enzymes and down-regulation of acyl-CoA oxidase in growth stages S3. The enzyme acyl-CoA oxidase has a function in breaking down specific types of fat molecules known as very long-chain fatty acids. It is responsible for lipid catabolism and the production of plant hormones. HADH and LCHAD, on the other hand, are enzymes that metabolize groups of fats known as medium-chain fatty acids and short-chain fatty acids. Totally, peroxisomal beta-oxidation has a profound impact on various physiological processes in plants, such as the conversion of triacylglyceride stores in oil seeds and regulation of plant lipid composition. It also plays a role in membrane lipid turnover, senescence, and starvation processes. Additionally, it helps synthesize fatty acid-derived hormones like jasmonic acid and indole-3-acetic acid, which influence a plant's stress response and growth regulation [67, 68].

### Glycerolipid metabolism (oeu00561)

In the glycerolipid metabolism pathway, glycerol-3-phosphate (G3P) as a precursor enter to reaction producing triacylglycerols (TAG). A review of the sources showed that G3P and dihydroxyacetone phosphate are key intermediates of oil biosynthesis [56]. Our results showed that the expression of glycerol kinase (EC: 2.7.1.30) increases in growth stages S2 and S3 where it can convert glycerol to G3P to initiate the main reactions to produce TAG (Fig. S8A and B - Additional file 10). Increased expression of glycerol kinase enzyme from stage S2 specifies that TAG production probably begins simultaneously with the production of fatty acids and continues in stage S3. The results of the present meta-analysis showed that the production of glycerol increases in the S3 stage by increasing the expression of enzymes aldehyde dehydrogenase (EC: 1.2.1.3) and alcohol dehydrogenase (EC: 1.1.1.2) so that the subsequent reactions that led to the production of TAG continue. The expression of these two enzymes increases in the production of acetyl coenzyme in the S3 stage as a compensatory mechanism, as described in the previous sections (Fig. 3). Both glycerol-3-phosphate acyltransferase and lysophosphatidic acid acyltransferase acetylated the G3P producing phosphatidic acid [69, 70]. The results of the meta-analysis show that the expression of lysophosphatidic acid acyltransferase (EC: 2.3.1.51) enzyme increased in growth stages S2 and S3. The phosphatidic acid is at the center of the TAG biosynthesis and different studies demonstrated its contribution to membrane lipid production, acyl editing, participation in response to biotic and abiotic stresses, endoplasmic reticulum lipid metabolism, vesicle trafficking, and cytoskeleton dynamics affect TAG production [39, 56]. For the direct production of diacylglycerols (DAG), which is the major precursor to TAG, the enzyme phosphatidate phosphatase (EC: 3.1.3.4) converted phosphatidic acid to DAG. The results of the present study show that the expression of phosphatidate phosphatase enzyme increased in the growth stage of S3. Several researches showed that lysophosphatidic acid acyltransferase, phosphatidate phosphatase and lysophosphatidylcholine acyltransferase are key enzymes in the biosynthesis pathway of TAG and membrane lipids that are effective in regulating phosphatidic acid homeostasis [56, 71, 72]. The results of the present study showed an up-regulation of lysophosphatidic acid acyltransferase enzyme in growth stages S2 and S3 and phosphatidate phosphatase enzyme in S3 stage. Therefore, phosphatidic acid and DAG are probably the main precursors of TAG production. Also, in the developmental stage of S3, the expression of the enzyme diacylglycerol kinase (EC: 2.7.1.107) increased where DAG is converted to phosphatidic acid. The diacylglycerol kinase is an enzyme that plays a critical role in lipid signaling, by catalyzing the phosphorylation of the DAG to phosphatidic acid, which is a crucial molecule in a plant’s metabolic network, leading to its response to various external stresses [73]. This indicated that the enzyme diacylglycerol kinase is effective in creating stability to produce DAG and phosphatidic acid. Research by Tan et al. [74] on the diacylglycerol kinase role in the freezing stress showed Arabidopsis mutants for this enzyme had lowest phosphatidic acid level in the stress condition and diacylglycerol kinase proteins are predominantly functional in converting DAG to phosphatidic acid upon freezing exposure. An important point in the DAG biosynthesis pathway is the composition of the fatty acids used in its structure, and the quality of the oil is determined by the affinity of the enzymes used in the G3P acetylation reactions [40]. The enzyme glycerol-3-phosphate acyltransferase, which performed the first step of G3P acetylation, preferred to use palmitoyl-CoA. Studies in olives showed that this enzyme prefers to use short-chain saturated fatty acids such as palmitoyl-CoA in the sn-1 than longer-chain saturated fatty acids [39]. Also, the decomposition of oil in olive drupes showed that linoleic acid binds the sn-1 and the enzyme glycerol-3-phosphate acyltransferase prefers to use linoleoyl-CoA as a precursor. Furthermore, this enzyme can use oleoyl-CoA if the share of oleoyl-CoA in the acetyl-CoA pool is higher [39, 40]. In most plants, especially olives in the sn-2 TAG position, the enzyme lysophosphatidic acid acyltransferase is highly preferred to use oleoyl-CoA as a precursor [40]. The results of the present study showed that the expression of this enzyme increases in two growth stages S2 and S3, which have high oleic acid production, and this enzyme could be considered as a key enzyme in the TAG biosynthesis pathway. Examination of the oil structure in olive fruits showed that this enzyme has a high selectivity for oleoyl-CoA and is inactivated in the presence of saturated fatty acids. This feature explained why TAGs of vegetable oils, including olive oil, do not contain saturated fatty acids in the sn-2 position [40]. Therefore, the quality of DAG produced depended on the choice of fatty acids in growth stage S2 and up-regulation of phosphatidate phosphatase in stage S3 influences increasing DAG production and then TAG.

**Fig. 5:**
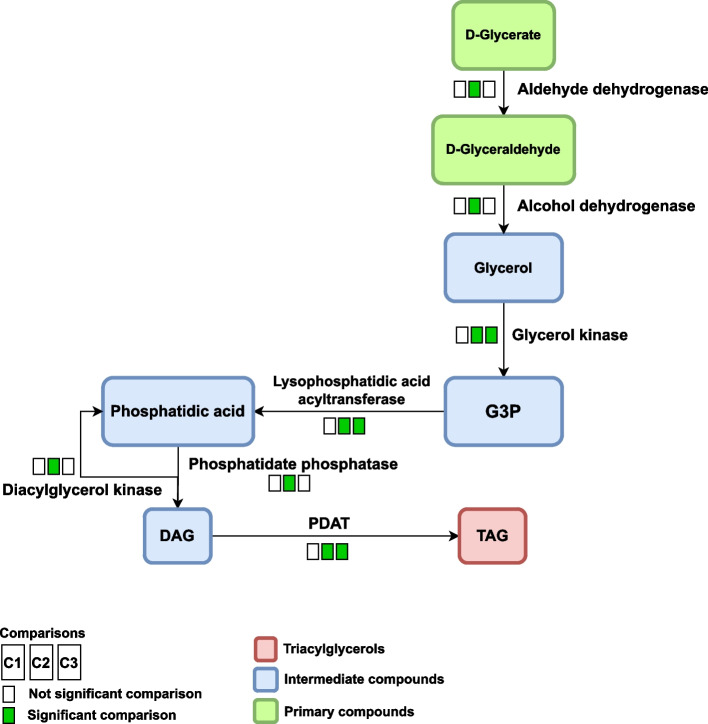
The independent TAG synthesis pathway from fatty acids through PDAT enzyme. The first square show the results of comparison 1 (*C1*: S1 *vs*. S2) and second and third squares show comparison 2 (*C2*: S1 *vs*. S3) and comparison 3 (*C3*: S2 *vs*. S3) respectively. For differential compounds, boxes in green represent up-regulated genes in each comparison and boxes in white represent no significant change

Two pathways are suggested to produce TAG from DAG. The first pathway is dependent on the presence of fatty acids and DAG is converted to TAG by the enzyme diacylglycerol acyltransferase (DGAT), while the second pathway is independent of the presence of fatty acids and DAG is transformed to TAG by the enzyme phospholipid:diacylglycerol acyltransferase (PDAT). The results of the present study showed that the expression of PDAT (EC: 2.3.1.158) increased in growth stages S2 and S3 and the conversion of DAG to TAG is produced from this pathway and is independent of fatty acids (Fig. 5). Therefore, in this pathway, the third TAG fatty acid could be supplied from phospholipids. If TAG is produced from a fatty acid-dependent pathway, the composition and type of the third fatty acid in the TAG structure will depend on the composition of the fatty acids in the acetyl-CoA pool and will reflect its composition [39, 40]. Examination of the pulp of growing olives by carbon 14 shows that the production of TAG from the fatty acid-dependent pathway is affected by environmental temperature and by increasing the temperature to more than 40 °C the expression of DGAT (EC: 2.3.1.20) decreased [39]. Therefore, TAG production from a pathway independent of fatty acids cannot depend on temperature and growth conditions and can be considered as a permanent pathway for TAG production. On the other hand, studies showed that DGAT can be a key enzyme in TAG accumulation, oil production and protein content in grain, and silencing the gene in tobacco reduces grain oil [56]. In a study conducted by Han et al. [75] DGAT3 was investigated in *Paeonia rockii* and was announced DGAT3 mainly expressed in the vegetative organs and also showed DGAT3 preference unsaturated fatty acid (α-linolenic acid and linoleic acid) in the last step of TAG biosynthesis. According to the present results, the production of TAG by the PDAT pathway probably occurred in the mesocarp and played an important role in the production of TAG and oil in the mesocarp. The results also showed that in the TAG degradation pathway, the expression of acylglycerol lipase (EC: 3.1.1.23) enzyme increases in S3 stage and converts monoacylglycerol to glycerol and free fatty acids.

### Terpenoid backbone biosynthesis (oeu00900)

The pathway of terpenoid backbone biosynthesis is one of the most important in which precursors of secondary metabolites present in olives are produced and this pathway can be introduced as a link between other pathways (monoterpenoid biosynthesis, diterpenoid biosynthesis, carotenoid biosynthesis, tocopherols biosynthesis, sesquiterpenoid and triterpenoid biosynthesis, and steroid biosynthesis). In the terpenoid backbone biosynthesis, there are two paths, the mevalonate pathway (MVA) and non-mevalonate pathway (MEP) (Fig. S9A and B - Additional file 11). In the MEP pathway, the results of the meta-analysis showed 1-deoxy-D-xylulose-5-phosphate synthase (DXS) enzymes (EC: 2.2.1.7) and 4-hydroxy-3-methylbut-2-en-1-yl diphosphate reductase (HMBPPR) (EC: 1.17.7.4) are down-regulated in the S2 and S3 and 2-C-methyl-D-erythritol 4-phosphate cytidylyltransferase (CDPMES) (EC: 2.7.7.60) is down-regulated in S3. The results of the meta-analysis also show that 4-diphosphocytidyl-2-C-methyl-D-erythritol kinase (CDPMEK) (EC: 2.7.1.148) is up-regulated in the growth stage of S2. On the other hand, CDPMEK enzyme, which, unlike other enzymes identified in S2 growth stage, increases its expression, can be considered as a new candidate in future studies. Therefore, according to the obtained results, the enzymes identified are active in the initial stages of growth in the MEP and with increasing growth stages, the expression of enzymes decreases. In a study performed by Bruno et al. [31], and in the research conducted by Alagna et al. [76] it was found that the expression of some genes in the MEP decreased sharply from the early stage of fruit growth and the amount of most secondary metabolites in different altitudes and cultivars had the highest rate in the early stages of growth.

In the MVA pathway, the expression of hydroxymethylglutaryl-CoA synthase (HMGCS) (EC: 2.3.3.10) increased up to a maximum in 130 days after flowering, after which its expression decreases. The results showed that in the MVA pathway the expression of two enzymes phosphomevalonate kinase (MVAPK) (EC: 2.7.4.2) and diphosphomevalonate decarboxylase (MVAPPD) (EC: 4.1.1.33) increased in the growth stage of S2 and S3 and Isopentenyl diphosphate (IPP) production increased. According to our results, HMGCS enzyme expression, which is located at the beginning of the MVA pathway, increased in S1 and S2. Most probably, it is a key enzyme in setting up MVA pathway and subsequent enzymes have their maximum activity in step S3. Increasing the expression of MVAK at all stages of development led to the production of mevalonic acid 5-phosphate known as the immediate precursor of IPP. Some previous studies have stated that there is no significant difference in the expression of this enzyme and others have shown that its expression increases at all stages of development [24, 76].

**Fig. 6:**
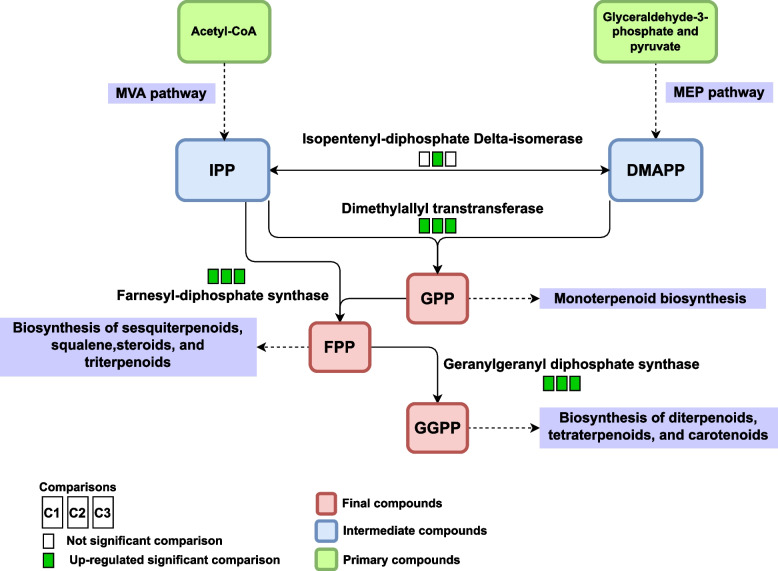
Production of precursors, pink color, for biosynthesis of important secondary metabolites. The first square show the results of comparison 1 (*C1*: S1 *vs*. S2) and second and third squares show comparison 2 (*C2*: S1 *vs*. S3) and comparison 3 (*C3*: S2 *vs*. S3) respectively. For differential compounds, boxes in green represent up-regulated genes in each comparison and boxes in white represent no significant change

Dimethylallyl diphosphate (DMAPP) is produced at the end of the MEP pathway, and IPP is produced at the end of the MVA pathway, and these components can turn to each other with isopentenyl-diphosphate Delta-isomerase (EC: 5.3.3.2) enzyme. This enzyme is known as the common enzyme between the two pathways and participating in the production of final products of both introduced pathways. The results of the meta-analysis showed that the expression of isopentenyl-diphosphate delta-isomerase enzyme increases in the growth stage of S3 which is consistent with the results of Alagna et al. [24]. Therefore, according to the obtained results, it can be said that the enzyme isopentenyl-diphosphate delta-isomerase has a key role in setting the next pathways and, by regulating the level of DMAPP and IPP, can activate the next pathways in the growth stage of S3. The results showed that the expression of dimethylallyl transtransferase (EC: 2.5.1.1) increased at all growth stages and converted DMAPP to Geranyl diphosphate (GPP). GPP entered the monoterpenoid biosynthesis pathway as a major precursor [77]. The results also showed that IPP can be converted to GPP by the enzyme dimethylallyltranstransferase. To produce other metabolites, IPP can be converted to Farnesyl diphosphate (FPP), and the biosynthesis of sesquiterpenoids, squalene, steroids, and triterpenoids will be performed [77]. The results showed that the expression of farnesyl-diphosphate synthase (EC 2.5.1.10) increased at all stages, and through its activity, IPP is converted to FPP. Subsequently, FPP is converted to GGPP by the enzyme geranylgeranyl diphosphate synthase (EC: 2.5.1.29) to produce diterpenoids, tetraterpenoids, and carotenoids. In other words, our results showed that to produce GGPP, first GPP is converted to FPP by the activity of farnesyl-diphosphate synthase and then to GGPP by the activity of the enzyme geranylgeranyl diphosphate synthase (Fig. 6). The results of the meta-analysis showed that the expression of geranylgeranyl diphosphate synthase increased at all stages of growth. It should be noted that the enzymes dimethylallyl transtransferase, farnesyl-diphosphate synthase, and geranylgeranyl diphosphate synthase function as an interconnected network and produce important precursors for other biosynthetic pathways. According to the results obtained in the studies of Alagna et al., (2009 and 2012) and the results obtained in the present study, the expression of enzymes introduced at all stages of growth is due to their role in the production of important downstream compounds such as oleuropein in olives. Also, GPP and GGPP, which are compounds derived from the MPE pathway in plastids, are used as substrates for the synthesis of monoterpene, diterpene, and tetraterpene, and FPP, which is a compound derived from the MVA pathway in the cytosol, is used as a substrate for triterpene and sesquiterpene biosynthesis [76, 78]. The results of Alagna et al. [76] confirmed the hypothesis that the MEP pathway is more involved in the biosynthesis of secoiridoids than the MVA pathway and primarily contributed to the biosynthesis of these compounds. Moreover, the MVA pathway began with the presence of acetyl-CoA, and the acetyl-CoA biosynthesis pathways mentioned in the previous sections can also affect the biosynthesis of secondary metabolites.

## Conclusions

According to the economic importance of olive oil, the quantity and quality of oil should be improved in parallel based on international standards in commercial cultivars. Technical variation in different experiments and limited number of biological replicates in RNA-seq researches could affect the reproducibility and the statistical power of the experiments. Meta-analysis of RNA-seq data can increase the speed of production of functional data related to the quality of olive oil and produce useful information. The present work would be an effective and useful step in reducing the time of cultivar screening by developing key genes relating to oil quality in olive breeding programs. The IDR index indicated the meta-analysis had good power to identify new differentially expressed genes and identified 155, 473, and 241 differential expressed genes for the first time respectively in the *C1 (S1 vs S2), C2 (S1 vs S3), and C3 (S2 vs S3)*. The identified key genes were investigated in the metabolic pathways related to oil quality and the identified metabolic pathways were also grouped into four groups according to the pathway of oil biosynthesis and the factors affecting its quality. The metabolic pathways that affecting the quality of oil have been examined independently confirming the earlier published results through meta-analysis and contributing to the reproducibility of the results. The galactose metabolism pathway can be further investigated as a source of carbon supply in olive fruit, and due to the increase in the expression of its genes in the S3, it can help to supply carbon in the final stage of growth when the fruit photosynthesis is reduced. Moreover, the production of acetyl-CoA in various ways in different growth stage shows the high efficiency of olive in the production of fatty acids. In the fatty acid biosynthesis pathway FabF and SAD have a critical role in oil quality and second identified pathway is more suitable for production of high oleic acid. Furthermore, the production of TAG by the PDAT probably occurred in the mesocarp and played an important role in the production of TAG and oil in the mesocarp. Also, the results of present study showed biosynthesis of phenolic compounds in olives in terms of growth stages might be in the opposite of the biosynthesis of fatty acids and there are the highest amount of phenolic compounds after fruit formation, while the biosynthesis of oil is at its lowest. The identified and confirmed genes by meta-analysis approach provided a suitable dataset to draw on for future studies in different scientific sectors. The present research can also play a role in cultivar selection by applying potential molecular markers developed in the here individuated key genes, reducing the duration of breeding programs, and helping the genome editing by introducing key and essential genes for the development of healthy compounds.

## Methods

### Search the databases and record experiments information

**Fig. 7:**
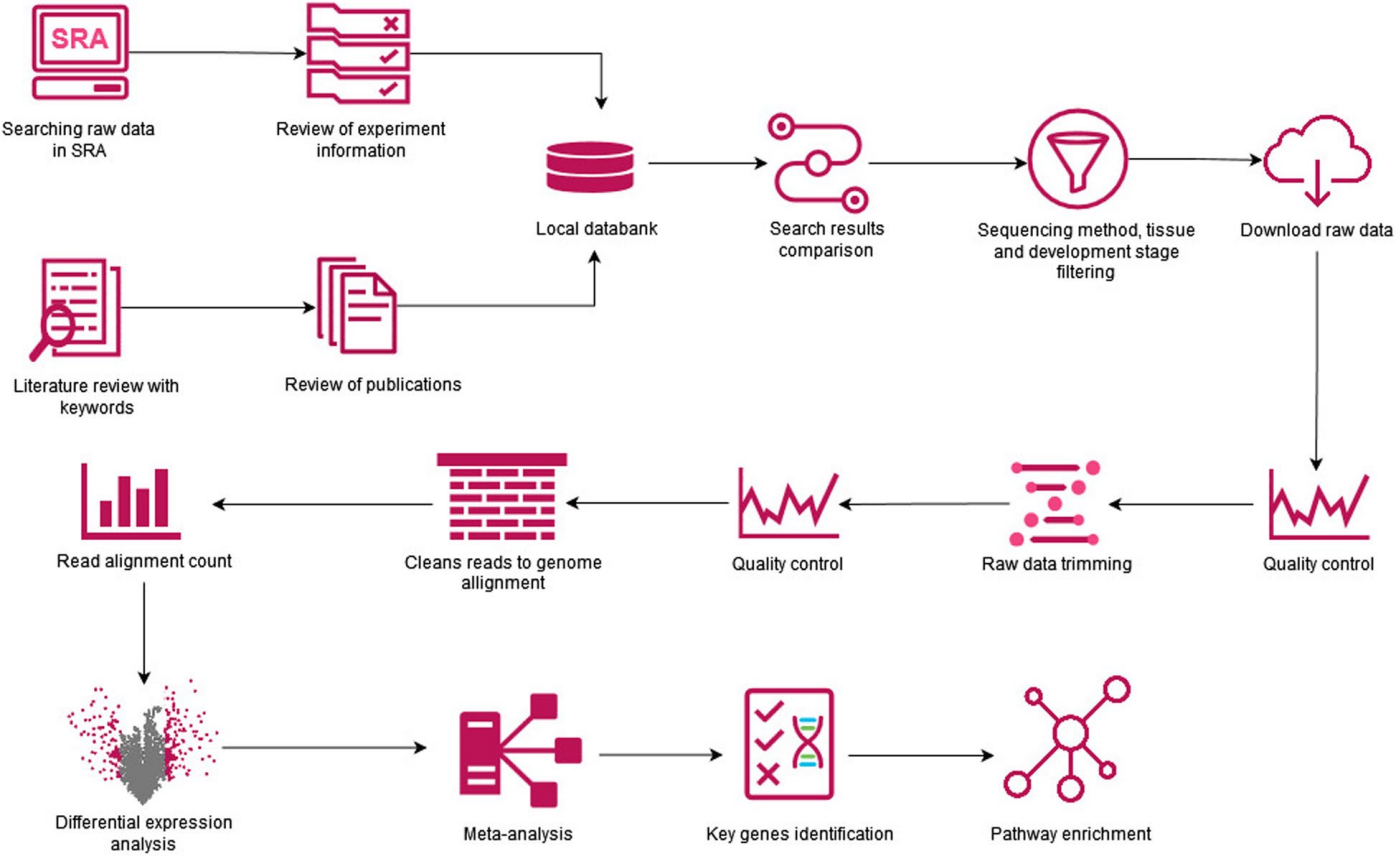
Steps of the meta-analysis for identification of key genes and their pathways related to olive oil quality

To search for raw sequencing data and publications related to the olive oil using RNA-seq method, the SRA database was investigated. The title and abstract of SRA project were examined in the first step, and the relevant data with olive RNA-seq were chosen. The obtained SRA description were reviewed independently for sample type and sequencing information. Then the information was recorded in a local dataset. In the second step, literature review was performed to increase the depth of the search and find more results. For this purpose, several keywords such as olive transcriptome, olive RNA-seq, olive sequencing, olive fruit, quality of olive oil, quantity of olive oil, oil biosynthesis, key genes in oil biosynthesis, phenolic or polyphenolic compounds were searched and related articles were selected. In SRA database, 126 run accessions and 26 related publications were found using literature review. After matching the search results of both strategies, the information of thirty nine experiments was recorded in our dataset (Additional file 12-Table S3). The selected experiments were filtered based on the sequencing platform (Illumina) and tissue (fruit), which resulted in nine experiments (Additional file 12-Table S4). Moreover, for meta-analysis the experiments with at least two developmental stages were selected (Additional file 12-Table S5). Finally, five experiments (PRJNA514943, PRJNA260808, PRJNA524768, PRJNA184000, and PRJNA638790) were selected and their raw sequencing data retrieved from ENA in fastq format (~ 77 gigabyte) (Fig. 7).

### Grouping the experiments

In the next step, the five selected experiments were grouped based on the developmental stage as reported in Galla et al. [26]. The experiments were divided into three groups (immature, semi-mature, and mature), and subsequent comparisons were performed among these groups. Experiments with the developmental stage less than 90 days after flowering (DAF) were considered in the immature group (X < 90; S1) and experiments with the growth stage between 90 and 130 DAF in the semi-mature group (90 ≤ X < 130; S2). Finally, experiments with a developmental stage of more than 130 DAF were in the mature stage (X ≥ 130; S3). To ensure grouping, the selected experiments were also grouped by principal component analysis and the t-SNE algorithm and glmPCA in R.

### Quality control and preprocessing of raw data

The quality control of raw Illumina RNA-seq reads was performed by FastQC software v0.11.8 (http://www.bioinformatics.babraham.ac.uk/projects/fastqc/). The raw reads were trimmed by Trimmomatic software v0.32 by discarding adaptors, ambiguous nucleotides, low-quality (< 20), and short-length reads (< 50 nt) for all the experiments. FastQC was utilized after each trimming to assess the properties of processed reads and to validate trimming efficiency. The data of PRJNA184000 was discarded since our standards parameters for quality could not be passed. The filtered, high-quality reads (four experiments) were used for mapping analyses.

### Mapping reads with the reference genome

The clean reads were mapped onto the *O. europaea* genome as the reference (OE6) (https://www.ncbi.nlm.nih.gov/assembly/GCA_900603015.1/) using the Hisat2 package. The aligned reads were sorted by position using the Samtools package. The read counts were calculated using the HTSeq package to estimate the count of uniquely mapped reads for each of the experiments. Finally, the read count matrix was used for the meta-analysis and differential expression analysis using edgeR.

### Meta-analysis

Meta-analysis was performed by metaRNASeq package [79]. Before performing a combination of *p*-values from each experiment, it is necessary to perform a differential expression analysis using the uniform method per study. We used the edgeR package [80, 81] to obtain *p*-values for differential analyses of each study independently. In the meta-analysis, the uniform distribution of raw *p*-values is a necessary assumption. Therefore, we checked this assumption by histograms of raw *p*-values for each experiment and confirmed the assumption for all studies. In the next step, the combination of *p*-values of experiments was performed by the Fisher method. Genes that showed contradictory different expressions in separate studies were removed from the expressed genes list [79]. Finally, a Venn diagram was drawn to show the genes identified in the meta-analysis in each experiment. It should be noted that the meta-analysis was performed separately to compare developmental stages (comparison 1 (*C1*): S1 *vs*. S2; *C2*: S1 *vs*. S3; *C3*: S2 *vs*. S3) as indicated above.

### Metabolic pathways analysis

The protein sequence of differentially expressed genes identified in the meta-analysis was used for pathway enrichment analysis by local KOBAS pipeline and in-house scripts. The KEGG pathways were examined and significant pathways were selected for more investigation. Related metabolic pathways were grouped according to their function and role in the biosynthesis of oil or its minor compounds was examined in detail. Also, selected metabolic pathways images were received from KEGG [82–84]. Finally, the effective genes in each metabolic pathway were identified and their functions were evaluated according to the research objectives.

### Supplementary Information

**Additional file 1: Figure S1.** Up- (A) and down-regulated (B) identified genes of the galactose metabolism pathway in the biosynthesis of raffinose family oligosaccharides.

**Additional file 2: Figure S2.** Up- (A) and down-regulated (B) identified genes of the glycolysis pathway in production of acetyl-CoA.

**Additional file 3: Figure S3.** Up- (A) and down-regulated (B) identified genes of the pyruvate pathway in production of acetyl-CoA.

**Additional file 4: Table S2.** The accession numbers of the identified up- and down-regulated genes in each of the investigated comparisons.

**Additional file 5: Table S1.** Identification of enriched genes in glycolysis and pentose phosphate pathways.

**Additional file 6: Figure S4.** Up- (A) and down-regulated (B) identified genes of the biotin metabolism pathway in production of acetyl-CoA carboxylase biotin carboxyl carrier protein.

**Additional file 7: Figure S5.** Up- (A) and down-regulated (B) identified genes in the Fatty acid biosynthesis pathway.

**Additional file 8: Figure S6.** Up- (A) and down-regulated (B) identified genes of the biosynthesis of unsaturated fatty acid pathway in production of linoleic acid.

**Additional file 9: Figure S7.** Up- (A) and down-regulated (B) identified genes in the Fatty acid degradation pathway.

**Additional file 10: Figure S8.** Up- (A) and down-regulated (B) identified genes in the glycerolipid metabolism pathway.

**Additional file 11: Figure S9.** Up- (A) and down-regulated (B) identified genes in the terpenoid backbone biosynthesis pathway.

**Additional file 12: Table S3.** The information of thirty nine experiments was recorded. **Table S4.** The selected experiments were filtered based on the sequencing platform (Illumina) and tissue (fruit). **Table S5.** The experiments with at least two developmental stages for meta-analysis.

**Additional file 13. **edgeR and metaRNASeq packages general code for differential expression and meta-analysis.

## Data Availability

The relevant data are reported in the main text and in additional file section. Further information and data could be available upon reasonable request to the corresponding author.
