# Supplementary material for: Meta-analysis of transcriptome reveals key genes relating to oil quality in olive
Source: BMC Genomics. 2023 Sep 22;24:566. doi: 10.1186/s12864-023-09673-y (PMC10517554; doi:10.1186/s12864-023-09673-y)
Supplement: Supplementary file 5 — Additional file 5: Table S1. Identification of enriched genes in glycolysis and pentose phosphate pathways. [file 12864_2023_9673_MOESM5_ESM.docx]

Table S1. Identification of enriched genes in glycolysis and pentose phosphate pathways

| **Pathway** | **Enriched genes** |
| --- | --- |
| Glycolysis | - Phosphoglucomutase (EC: 5.4.2.2) - 6-phosphofructokinase (EC: 2.7.1.90 and EC: 2.7.1.11) - Fructose-bisphosphate aldolase (EC: 4.1.2.13) - Triose-phosphate isomerase (EC: 5.3.1.1) - Glyceraldehyde-3-phosphate dehydrogenase (EC 1.2.1.12) - Phosphoglycerate mutase (EC: 5.4.2.11 and EC: 5.4.2.12) - Pyruvate kinase (EC: 2.7.1.40) - Plastidial pyruvate dehydrogenase (EC: 1.2.4.1 and EC: 2.3.1.12) - Pyruvate decarboxylase (EC: 4.1.1.1) - Alcohol dehydrogenase (EC: 1.1.1.2 and EC:1.1.1.1) - Aldehyde dehydrogenase (EC: 1.2.1.3) - Acetyl-CoA synthetase (EC: 6.2.1.1) - Dihydrolipoamide dehydrogenase (EC:1.8.1.4) - Hexokinase (EC:2.7.1.1) - Fructose-1,6-bisphosphatase I (EC:3.1.3.11) - Phosphoglycerate kinase (EC:2.7.2.3) - L-lactate dehydrogenase (EC:1.1.1.27) |
| Pentose phosphate | - glucose-6-phosphate 1-dehydrogenase (EC:1.1.1.49 and 1.1.1.363) - Diphosphate-dependent phosphofructokinase (EC:2.7.1.90) - 6-phosphofructokinase 1 (EC:2.7.1.11) - Fructose-bisphosphate aldolase, class I (EC:4.1.2.13) - Fructose-1,6-bisphosphatase I (EC:3.1.3.11) - Transketolase (EC:2.2.1.1) - Ribose 5-phosphate isomerase A (EC:5.3.1.6) - Phosphoglucomutase (EC:5.4.2.2) - 6-phosphogluconolactonase (EC:3.1.1.31) - Ribulose-phosphate 3-epimerase (EC:5.1.3.1) |
