# Supplementary material for: Meta-analysis of transcriptome reveals key genes relating to oil quality in olive
Source: BMC Genomics. 2023 Sep 22;24:566. doi: 10.1186/s12864-023-09673-y (PMC10517554; doi:10.1186/s12864-023-09673-y)
Supplement: Supplementary file 8 — Additional file 8: Figure S6. Up- (A) and down-regulated (B) identified genes of the biosynthesis of unsaturated fatty acid pathway in production of linoleic acid. [file 12864_2023_9673_MOESM8_ESM.docx]

**A**


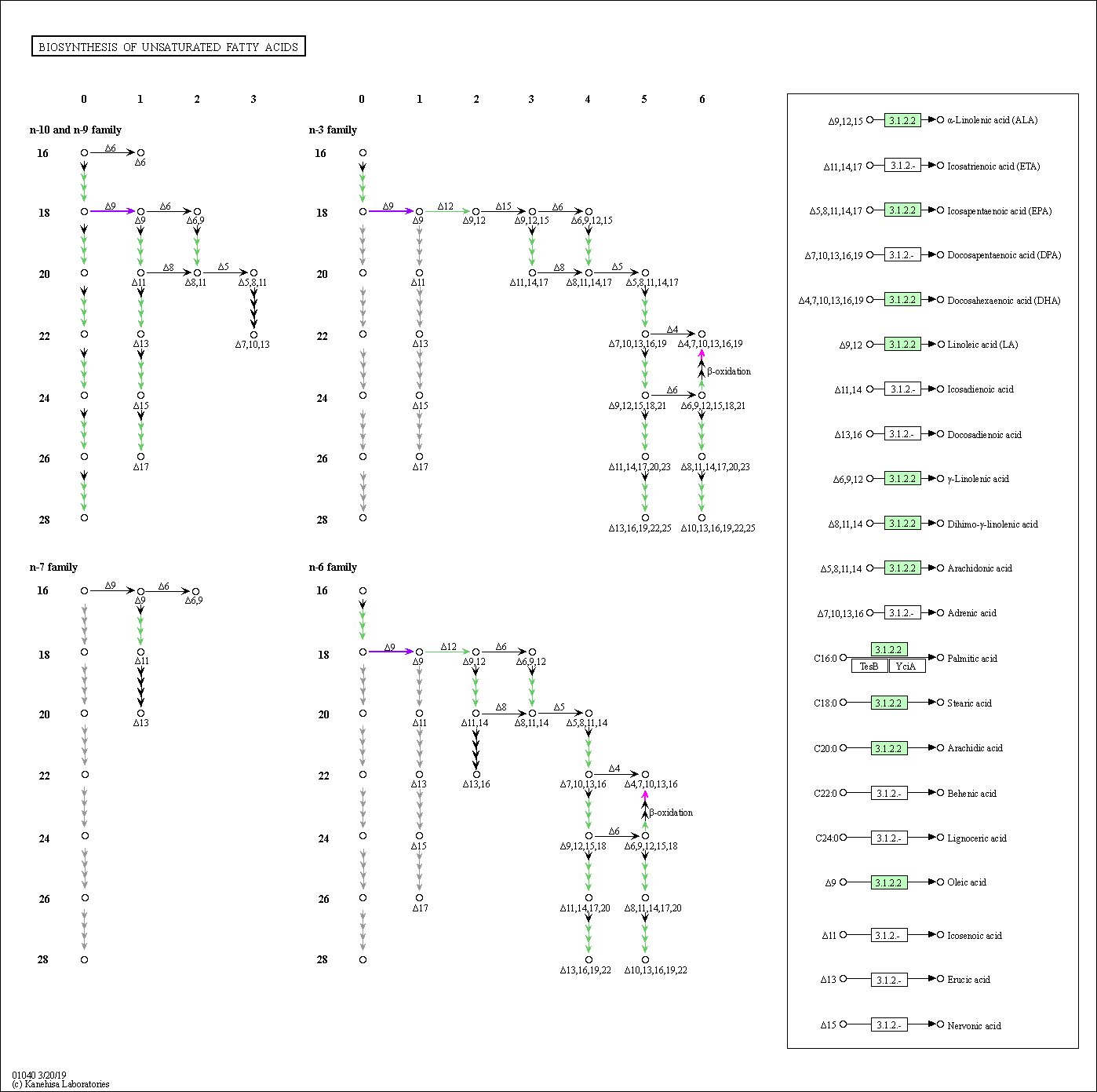


**B**


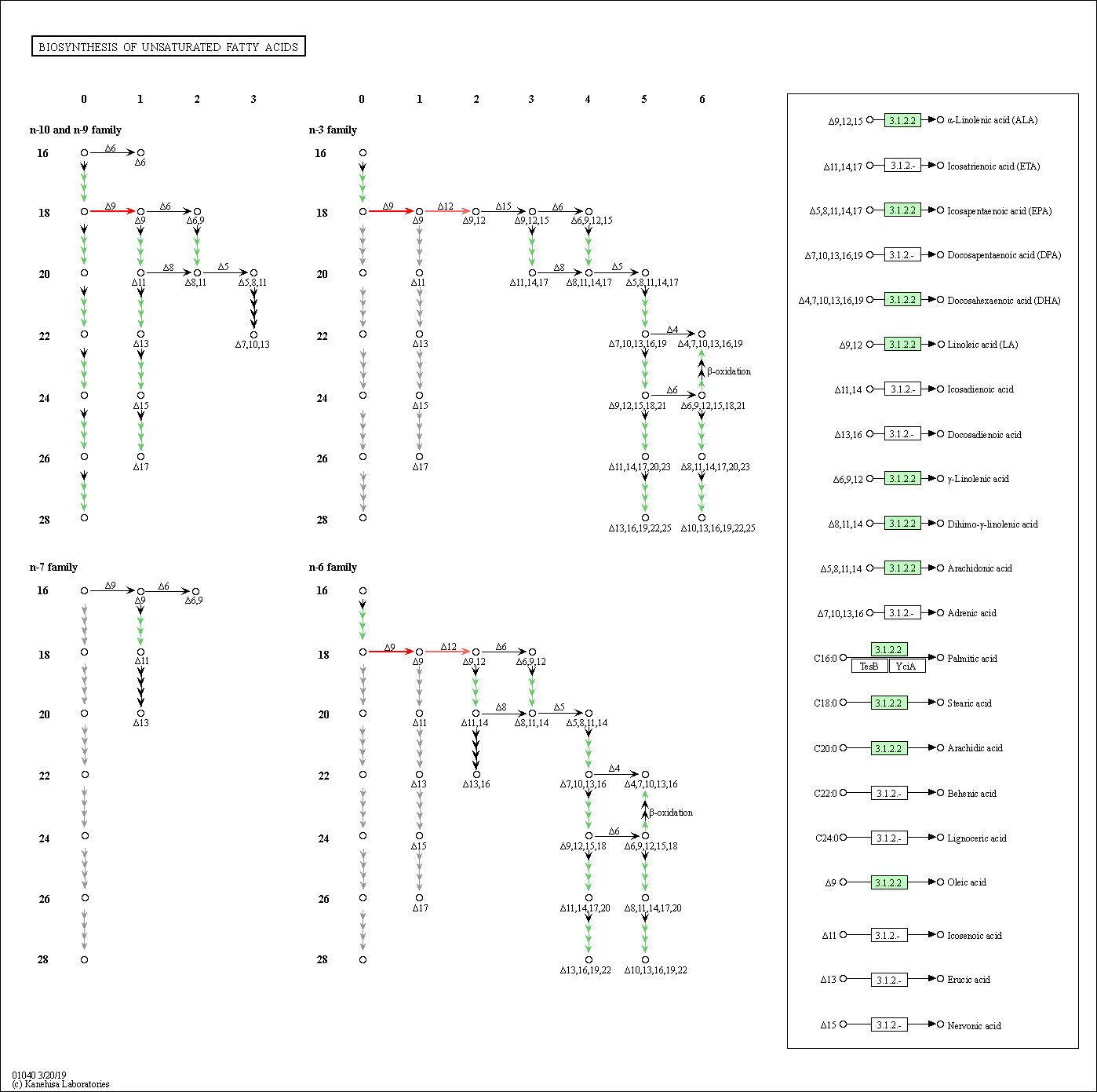


**Figure S6.** Up- (A) and down-regulated (B) identified genes of the biosynthesis of unsaturated fatty acid pathway in production of linoleic acid

**The colors legend:** The genes that showed expression changes in one comparison are marked with blue (*C1: S1 vs S2*), red (*C2: S1 vs S3*) and yellow (*C3: S2 vs S3*) colors and the genes that showed expression changes in two comparisons are marked with purple (*C1: S1 vs S2 and C2: S1 vs S3*), orange (*C1: S1 vs S2 and C3: S2 vs S3*) and pink (*C2: S1 vs S3 and C3: S2 vs S3*) colors and if a gene showed expression changes in all comparisons (*C1: S1 vs S2, C2: S1 vs S3, and C3: S2 vs S3*) are highlighted in light red.
